# Bis(3-hy­droxy­methyl­anilinium) hexa­chloridostannate(IV)

**DOI:** 10.1107/S1600536811007252

**Published:** 2011-03-02

**Authors:** Sofiane Bouacida, Habiba Kechout, Ratiba Belhouas, Hocine Merazig, Thierry Roisnel

**Affiliations:** a Unité de Recherche de Chimie de l’Environnement et Moléculaire Structurale, CHEMS, Université Mentouri-Constantine, 25000 Algeria; b Département Sciences de la Matière, Facult des Sciences Exactes et Sciences de la Nature et de la Vie, Université Larbi Ben M’hidi, Oum El Bouaghi 04000, Algeria; c Centre de Difractométrie X, UMR 6226 CNRS Unité Sciences Chimiques de Rennes, Université de Rennes I, 263 Avenue du Général Leclerc, 35042 Rennes, France

## Abstract

In the title compound, (C_7_H_10_NO)_2_[SnCl_6_], the Sn^IV^ atom, located on an inversion center, exists in an octa­hedral coordination environment. The crystal structure exhibits alternating organic and inorganic layers parallel to (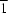
01). The cations and anions are linked *via* inter­molecular N—H⋯O, N—H⋯Cl and O—H⋯Cl hydrogen bonds. Additional stabilization is provided by π–π stacking inter­actions between the benzene rings of the cations [centroid–centroid distances = 3.6962 (15) and 3.9340 (15) Å].

## Related literature

For related structures of similar monoprotonated amines or imines, see: Bouacida (2008 ▶); Bouacida *et al.* (2005*a* ▶,*b* ▶,*c* ▶, 2009 ▶); Rademeyer (2004*a* ▶,*b* ▶). For a description of the Cambridge Structural Database, see: Allen (2002 ▶).
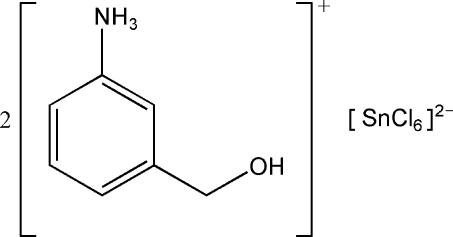


## Experimental

### 

#### Crystal data

(C_7_H_10_NO)_2_[SnCl_6_]
*M* *_r_* = 579.73
Monoclinic, 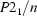

*a* = 7.4785 (11) Å
*b* = 11.2959 (16) Å
*c* = 12.6153 (18) Å
β = 105.989 (5)°
*V* = 1024.5 (3) Å^3^
*Z* = 2
Mo *K*α radiation
μ = 2.04 mm^−1^
*T* = 100 K
0.20 × 0.18 × 0.16 mm

#### Data collection

Bruker APEXII CCD diffractometer
Absorption correction: multi-scan (*SADABS*; Sheldrick, 1996 ▶) *T*_min_ = 0.423, *T*_max_ = 0.693
5915 measured reflections
2279 independent reflections
2027 reflections with *I* > 2σ(*I*)
*R*_int_ = 0.038

#### Refinement

*R*[*F* ^2^ > 2σ(*F* ^2^)] = 0.025
*wR*(*F* ^2^) = 0.058
*S* = 1.07
2279 reflections
117 parameters
H-atom parameters constrained
Δρ_max_ = 0.63 e Å^−3^
Δρ_min_ = −1.27 e Å^−3^

### 

Data collection: *APEX2* (Bruker, 2007 ▶); cell refinement: *SAINT* (Bruker, 2007 ▶); data reduction: *SAINT*; program(s) used to solve structure: *SIR2002* (Burla *et al.*, 2003 ▶); program(s) used to refine structure: *SHELXL97* (Sheldrick, 2008 ▶); molecular graphics: *ORTEP-3* (Farrugia, 1997 ▶) and *DIAMOND* (Brandenburg & Berndt, 1999 ▶); software used to prepare material for publication: *WinGX* (Farrugia, 1999 ▶).

## Supplementary Material

Crystal structure: contains datablocks global, I. DOI: 10.1107/S1600536811007252/hy2408sup1.cif

Structure factors: contains datablocks I. DOI: 10.1107/S1600536811007252/hy2408Isup2.hkl

Additional supplementary materials:  crystallographic information; 3D view; checkCIF report

## Figures and Tables

**Table 1:** Hydrogen-bond geometry (Å, °)

*D*—H⋯*A*	*D*—H	H⋯*A*	*D*⋯*A*	*D*—H⋯*A*
O1—H1⋯Cl2	0.82	2.70	3.438 (2)	151
O1—H1⋯Cl3	0.82	2.79	3.370 (2)	130
N1—H1*A*⋯Cl3^i^	0.89	2.64	3.298 (2)	131
N1—H1*B*⋯O1^ii^	0.89	1.83	2.721 (3)	175
N1—H1*C*⋯Cl1^iii^	0.89	2.71	3.338 (2)	128
N1—H1*C*⋯Cl2^iii^	0.89	2.57	3.304 (2)	140

Symmetry codes: (i) 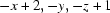; (ii) 
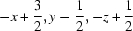
; (iii) 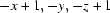.

**Fig. 1.**
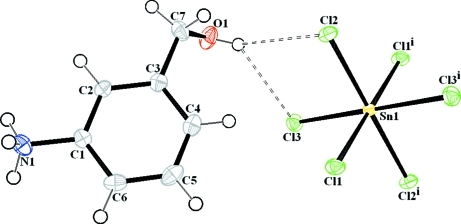
Molecular structure of the title compound. Displacement ellipsoids are drawn at the 50% probability level. Hydrogen bonds are shown as dashed lines. [Symmetry code: (i) 1-x, 1-y, 1-z.]

**Fig. 2.**
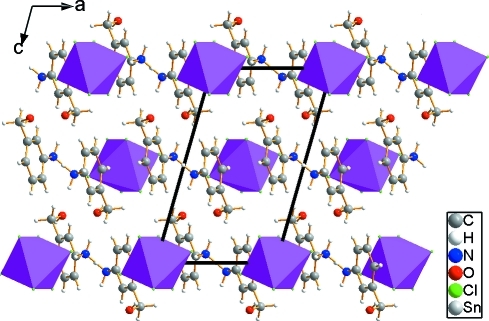
A diagram of the layered crystal packing in the title compound, viewed down the b axis, showing alternating layers of octahedral anions and cations.

**Fig. 3.**
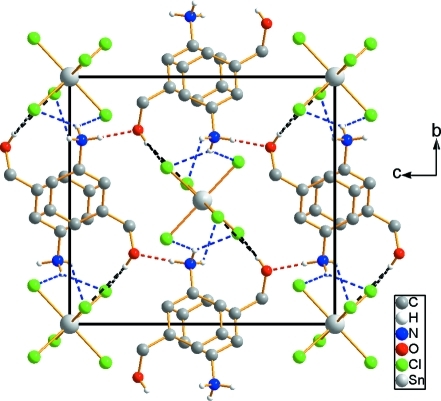
Crystal packing of the title compound, viewed down the a axis, showing hydrogen bonds (dashed lines). H atoms not involved in hydrogen bonds have been omitted for clarity.

## supplementary crystallographic information

### Comment

The title compound was prepared as part of our ongoing studies of hydrogen-bonding interactions in the crystal structures of protonated amines (Bouacida, 2008; Bouacida *et al.*, 2009).

In the title compound (Fig. 1), all bond distances and angles are within the ranges of accepted values (CSD, Allen, 2002). The amino N atom is protonated as in the other amines and imines (Bouacida *et al.*, 2005*a*,b,c; Rademeyer, 2004*a*,b). The Sn^IV^ atom is six-coordinated with six Cl atoms, located on an inversion center, forming a slightly distorted octahedral geometry. The crystal structure can be described as alternating layers of [SnCl_6_]^2-^ comlpex anions and 3-hydroxymethylanilinium cations parallel to (1 0 1) (Fig. 2). In the crystal, the components of the structure are linked *via* intermolecular N—H···O, N—H···Cl and O—H···Cl hydrogen bonds (Table 1, Fig. 3). Additional stabilization is provided by π–π stacking interactions (Table 2). These interactions link the cations and anions together, reinforcing the cohesion of the ionic structure.

### Experimental

Crystals of the title compound were grown from an aqueous solution that was obtained by dissolving SnCl_2_ (1 mmol) and 3-aminophenylmethanol (2 mmol) in hydrochloric acid. The solution was slowly evaporated to dryness for a couple of weeks. Some colorless crystals were carefully isolated under polarizing microscope for X-ray diffraction analysis.

### Refinement

H atoms were located in difference Fourier maps but introduced in calculated positions and treated as riding on their parent atoms, with C—H = 0.93 and 0.97, N—H = 0.89, and O—H = 0.82 Å, and *U*_iso_(H) = 1.2*U*_eq_(C) or 1.5*U*_eq_(N, O).

### Crystal data

**Table:**

(C_7_H_10_NO)_2_[SnCl_6_]	*F*(000) = 572
*M**_r_* = 579.73	*D*_x_ = 1.879 Mg m^−^^3^
Monoclinic, *P*2_1_/*n*	Mo *K*α radiation, λ = 0.71073 Å
*a* = 7.4785 (11) Å	Cell parameters from 3840 reflections
*b* = 11.2959 (16) Å	θ = 3.4–27.4°
*c* = 12.6153 (18) Å	µ = 2.04 mm^−^^1^
β = 105.989 (5)°	*T* = 100 K
*V* = 1024.5 (3) Å^3^	Block, colorless
*Z* = 2	0.20 × 0.18 × 0.16 mm

### Data collection

**Table:**

Bruker APEXII CCD diffractometer	2027 reflections with *I* > 2σ(*I*)
graphite	*R*_int_ = 0.038
φ and ω scans	θ_max_ = 27.5°, θ_min_ = 2.5°
Absorption correction: multi-scan (*SADABS*; Sheldrick, 1996)	*h* = −9→5
*T*_min_ = 0.423, *T*_max_ = 0.693	*k* = −10→14
5915 measured reflections	*l* = −14→16
2279 independent reflections	

### Refinement

**Table:**

Refinement on *F*^2^	Primary atom site location: structure-invariant direct methods
Least-squares matrix: full	Secondary atom site location: difference Fourier map
*R*[*F*^2^ > 2σ(*F*^2^)] = 0.025	Hydrogen site location: inferred from neighbouring sites
*wR*(*F*^2^) = 0.058	H-atom parameters constrained
*S* = 1.07	*w* = 1/[σ^2^(*F*_o_^2^) + (0.0194*P*)^2^ + 0.1921*P*] where *P* = (*F*_o_^2^ + 2*F*_c_^2^)/3
2279 reflections	(Δ/σ)_max_ = 0.001
117 parameters	Δρ_max_ = 0.63 e Å^−^^3^
0 restraints	Δρ_min_ = −1.27 e Å^−^^3^

### Fractional atomic coordinates and isotropic or equivalent isotropic displacement parameters (Å^2^)

**Table:**

	*x*	*y*	*z*	*U*_iso_*/*U*_eq_	
C1	0.8165 (3)	−0.12498 (19)	0.46444 (19)	0.0181 (5)	
C2	0.7132 (3)	−0.06992 (19)	0.36967 (18)	0.0170 (5)	
H2	0.6918	−0.1075	0.3017	0.02*	
C3	0.6412 (3)	0.0428 (2)	0.37701 (19)	0.0186 (5)	
C4	0.6736 (4)	0.0954 (2)	0.48072 (19)	0.0216 (5)	
H4	0.6246	0.17	0.4867	0.026*	
C5	0.7765 (4)	0.0391 (2)	0.5742 (2)	0.0249 (6)	
H5	0.7973	0.0761	0.6424	0.03*	
C6	0.8502 (3)	−0.0738 (2)	0.56723 (19)	0.0207 (5)	
H6	0.9197	−0.1128	0.63	0.025*	
C7	0.5266 (4)	0.1037 (2)	0.2755 (2)	0.0228 (5)	
H7A	0.5162	0.0529	0.2121	0.027*	
H7B	0.4023	0.1172	0.2826	0.027*	
N1	0.8919 (3)	−0.24370 (17)	0.45477 (16)	0.0219 (4)	
H1A	1.005	−0.2499	0.5014	0.033*	
H1B	0.8986	−0.2547	0.3861	0.033*	
H1C	0.8176	−0.2982	0.471	0.033*	
O1	0.6072 (3)	0.21527 (14)	0.25759 (14)	0.0284 (4)	
H1	0.5691	0.2678	0.2907	0.043*	
Cl1	0.54335 (8)	0.34435 (5)	0.63534 (4)	0.01892 (13)	
Cl2	0.29979 (8)	0.36720 (5)	0.36576 (4)	0.01874 (14)	
Cl3	0.76854 (9)	0.43264 (5)	0.44207 (5)	0.02107 (14)	
Sn1	0.5	0.5	0.5	0.01371 (8)	

### Atomic displacement parameters (Å^2^)

**Table:**

	*U*^11^	*U*^22^	*U*^33^	*U*^12^	*U*^13^	*U*^23^
C1	0.0175 (13)	0.0183 (11)	0.0200 (12)	0.0004 (10)	0.0073 (10)	0.0020 (9)
C2	0.0181 (13)	0.0167 (11)	0.0162 (11)	−0.0023 (10)	0.0049 (10)	−0.0015 (9)
C3	0.0171 (13)	0.0174 (11)	0.0218 (12)	−0.0041 (10)	0.0062 (10)	−0.0006 (10)
C4	0.0213 (13)	0.0194 (11)	0.0268 (13)	−0.0046 (11)	0.0112 (11)	−0.0042 (10)
C5	0.0268 (15)	0.0302 (13)	0.0207 (13)	−0.0091 (12)	0.0118 (11)	−0.0071 (11)
C6	0.0189 (13)	0.0266 (12)	0.0159 (12)	−0.0060 (11)	0.0037 (10)	0.0033 (10)
C7	0.0195 (14)	0.0179 (11)	0.0298 (14)	0.0007 (10)	0.0047 (11)	0.0037 (10)
N1	0.0216 (12)	0.0213 (10)	0.0240 (11)	0.0028 (9)	0.0083 (9)	0.0064 (8)
O1	0.0455 (13)	0.0152 (8)	0.0273 (10)	−0.0035 (9)	0.0144 (9)	−0.0013 (7)
Cl1	0.0239 (3)	0.0162 (3)	0.0145 (3)	−0.0011 (2)	0.0017 (2)	0.0028 (2)
Cl2	0.0234 (3)	0.0171 (3)	0.0138 (3)	−0.0036 (2)	0.0019 (2)	−0.0007 (2)
Cl3	0.0195 (3)	0.0229 (3)	0.0219 (3)	0.0023 (2)	0.0074 (2)	0.0003 (2)
Sn1	0.01617 (13)	0.01257 (12)	0.01161 (13)	0.00006 (8)	0.00251 (9)	−0.00002 (8)

### Geometric parameters (Å, °)

**Table:**

C1—C6	1.378 (3)	C6—H6	0.93
C1—C2	1.380 (3)	C7—O1	1.441 (3)
C1—N1	1.473 (3)	C7—H7A	0.97
C2—C3	1.396 (3)	C7—H7B	0.97
C2—H2	0.93	N1—H1A	0.89
C3—C4	1.396 (3)	N1—H1B	0.89
C3—C7	1.498 (3)	N1—H1C	0.89
C4—C5	1.374 (4)	O1—H1	0.82
C4—H4	0.93	Sn1—Cl1	2.4097 (6)
C5—C6	1.401 (3)	Sn1—Cl2	2.4419 (6)
C5—H5	0.93	Sn1—Cl3	2.4402 (6)
			
C6—C1—C2	122.7 (2)	H7A—C7—H7B	107.9
C6—C1—N1	119.0 (2)	C1—N1—H1A	109.5
C2—C1—N1	118.3 (2)	C1—N1—H1B	109.5
C1—C2—C3	119.2 (2)	H1A—N1—H1B	109.5
C1—C2—H2	120.4	C1—N1—H1C	109.5
C3—C2—H2	120.4	H1A—N1—H1C	109.5
C4—C3—C2	118.7 (2)	H1B—N1—H1C	109.5
C4—C3—C7	121.1 (2)	C7—O1—H1	109.5
C2—C3—C7	120.2 (2)	Cl1—Sn1—Cl1^i^	180
C5—C4—C3	121.3 (2)	Cl1—Sn1—Cl3^i^	88.65 (2)
C5—C4—H4	119.4	Cl1^i^—Sn1—Cl3^i^	91.35 (2)
C3—C4—H4	119.4	Cl1—Sn1—Cl3	91.35 (2)
C4—C5—C6	120.3 (2)	Cl1^i^—Sn1—Cl3	88.65 (2)
C4—C5—H5	119.8	Cl3^i^—Sn1—Cl3	180
C6—C5—H5	119.8	Cl1—Sn1—Cl2^i^	91.13 (2)
C1—C6—C5	117.9 (2)	Cl1^i^—Sn1—Cl2^i^	88.87 (2)
C1—C6—H6	121.1	Cl3^i^—Sn1—Cl2^i^	89.93 (2)
C5—C6—H6	121.1	Cl3—Sn1—Cl2^i^	90.07 (2)
O1—C7—C3	111.7 (2)	Cl1—Sn1—Cl2	88.87 (2)
O1—C7—H7A	109.3	Cl1^i^—Sn1—Cl2	91.13 (2)
C3—C7—H7A	109.3	Cl3^i^—Sn1—Cl2	90.07 (2)
O1—C7—H7B	109.3	Cl3—Sn1—Cl2	89.93 (2)
C3—C7—H7B	109.3	Cl2^i^—Sn1—Cl2	180.000 (19)
			
C6—C1—C2—C3	0.8 (4)	C3—C4—C5—C6	−0.6 (4)
N1—C1—C2—C3	−179.7 (2)	C2—C1—C6—C5	−0.4 (4)
C1—C2—C3—C4	−1.1 (3)	N1—C1—C6—C5	−179.8 (2)
C1—C2—C3—C7	−179.2 (2)	C4—C5—C6—C1	0.2 (4)
C2—C3—C4—C5	1.0 (4)	C4—C3—C7—O1	60.8 (3)
C7—C3—C4—C5	179.1 (2)	C2—C3—C7—O1	−121.2 (2)

Symmetry codes: (i) −*x*+1, −*y*+1, −*z*+1.

### Hydrogen-bond geometry (Å, °)

**Table:**

*D*—H···*A*	*D*—H	H···*A*	*D*···*A*	*D*—H···*A*
O1—H1···Cl2	0.82	2.70	3.438 (2)	151
O1—H1···Cl3	0.82	2.79	3.370 (2)	130
N1—H1A···Cl3^ii^	0.89	2.64	3.298 (2)	131
N1—H1B···O1^iii^	0.89	1.83	2.721 (3)	175
N1—H1C···Cl1^iv^	0.89	2.71	3.338 (2)	128
N1—H1C···Cl2^iv^	0.89	2.57	3.304 (2)	140

Symmetry codes: (ii) −*x*+2, −*y*, −*z*+1; (iii) −*x*+3/2, *y*−1/2, −*z*+1/2; (iv) −*x*+1, −*y*, −*z*+1.

### Table 2 Table 2. π–π stacking interactions (Å,°)

**Table:**

*Cg*I	*Cg*J	*Cg*I···*Cg*J	β	*Cg*I···*P*J	slippage
*Cg*1	*Cg*1^i^	3.6962 (15)	25.82	-3.3272 (10)	1.610
*Cg*1	*Cg*1^ii^	3.9340 (15)	29.64	3.4194 (10)	1.945

Symmetry codes: (i) -x,-y,1-z; (ii) 1-x,-y,1-z. Notes: *Cg*1 is the centroid of the C1–C6 ring; *Cg*I···*Cg*J is the distance between the centroids; *Cg*I···*P*J is the perpendicular distance of *Cg*I on ring plane J; β is the angle between the vector *Cg*I—*Cg*J and the normal to ring plane I; slippage is the distance between *Cg*I and the projection of *Cg*J on ring plane I.
